# The Role of Dairy Products and Milk in Adolescent Obesity: Evidence from Hong Kong’s “Children of 1997” Birth Cohort

**DOI:** 10.1371/journal.pone.0052575

**Published:** 2012-12-20

**Authors:** Shi Lin Lin, Marie Tarrant, Lai Ling Hui, Man Ki Kwok, Tai Hing Lam, Gabriel M. Leung, C. Mary Schooling

**Affiliations:** 1 Lifestyle and Life Course Epidemiology Group, School of Public Health, Li Ka Shing Faculty of Medicine, The University of Hong Kong, Hong Kong SAR, People’s Republic of China; 2 School of Nursing, Li Ka Shing Faculty of Medicine, The University of Hong Kong, Hong Kong SAR, People’s Republic of China; 3 City University of New York School of Public Health at Hunter College, New York, United States of America; College of Tropical Agriculture and Human Resources, University of Hawaii, United States of America

## Abstract

**Background:**

Observational studies, mainly from Western populations, suggest dairy consumption is inversely associated with adiposity. However, in these populations the intake range is limited and both diet and obesity may share social patterning. Evidence from non-Western developed settings with different social patterning, is valuable in distinguishing whether observed associations are biologically mediated or socially confounded.

**Objective:**

To examine the associations of milk or other dairy product consumption with adolescent obesity.

**Methods:**

We used multivariable linear regression models to examine the associations of milk or other dairy product consumption, obtained from a food frequency questionnaire, at 11 years with body mass index (BMI) *z*-scores at 13 years and waist hip ratio (WHR) at 11 years, in 5,968 adolescents from a Chinese birth cohort, comprising 88% of births in April and May 1997. We used multiple imputation for missing exposures and confounders.

**Results:**

Only 65.7% regularly consumed milk and 72.4% other dairy products. Milk and other dairy product consumption was positively associated with socio-economic position but not with BMI *z*-score or WHR, with or without adjustment for sex, mother’s birthplace, parental education, physical activity and other food consumption.

**Conclusions:**

The lack of association of milk and other dairy product consumption with adiposity in a non-Western setting was not consistent with the majority of evidence from Western settings. Observed anti-obesigenic effects in Western settings may be due to socially patterned confounding.

## Introduction

Child and adolescent obesity has increased rapidly and has become a global epidemic. Lifestyle factors such as diet play an important role. Dairy products and milk consumption have been observed inversely associated with body mass or fat mass in cross-sectional studies of children and adolescents. [1], [2] However, a recent review of prospective cohort studies in Western settings found inconsistent associations of dairy product consumption with overweight or obesity. [3] In the 10 studies concerning children and adolescents, 4 studies found negative association of dairy product consumption with obesity, [4], [5], [6], [7] 5 found no association [8], [9], [10], [11], [12] and one found a positive association. [13] Limited evidence from randomized controlled trials (RCTs) in adolescent girls did not find an inverse effect. [14], [15], [16] However, most of RCTs were originally designed to examine the effect on bone mineral density, and may not have been powered to detect effects on body fat.

RCTs are a vital source of high-quality evidence to guide policy and practice. Nevertheless, given the equivocal evidence and the possibility of uncontrolled and uncontrollable residual confounding in the observational studies generating these hypotheses, it is important to validate such evidence in other settings or social laboratories before proceeding to trials, particularly in children. Unlike in Western developed countries, childhood and adolescent obesity is less clearly socially patterned in China. [17], [18] Dairy products are not a traditional part of the Chinese diet, so there is a much wider range of consumption than in many Western societies, with many consuming little or none at all. [19] Moreover, with westernization dairy products are heavily promoted and increasingly consumed in China. There is now a key window of opportunity to identify the role of dairy products and intervene, as necessary, before dietary habits change irrevocably with westernization. We used a large, contemporary Hong Kong Chinese birth cohort “Children of 1997”, from a region with a recent history of economic development, to assess the association of dairy product consumption prospectively with adolescent obesity.

## Methods

### Ethics Statement

Ethical approval was obtained from the University of Hong Kong-Hospital Authority Hong Kong West Cluster, Joint Institutional Review Board and the Ethics Committee of the Department of Health, Government of the Hong Kong SAR.

### Source of Data

The Hong Kong “Children of 1997” birth cohort is a Chinese birth cohort (*n* = 8,327) that covered 88.0% of all births from April 1, 1997 to May 31, 1997. The study was initially established to investigate the effect of second hand smoke exposure on infant health. [20], [21] Families were recruited at the first postnatal visit to any of the 49 Maternal and Child Health Centers (MCHCs) in Hong Kong, [20] which parents of all newborns are encouraged to attend for free postnatal care, developmental checks and vaccinations until the age of five years. Baseline characteristics were obtained at recruitment using a self-administered questionnaire in Chinese and included socio-demographic information and birth characteristics. Passive follow-up via record linkage was instituted in 2005 to obtain: (i) weight and height from birth to 5 years from the MCHCs (96% success); (ii) annual measurements of weight and height (age 6–7 years onwards) and bi-annual assessments of pubertal status from the Student Health Service, Department of Health, which provides free annual check-ups for all school students; [22] and (iii) death records from the Death Registry. Active follow-up via direct contact was instituted in 2007. A postal survey (Survey I) including questions on child’s lifestyle was sent in July 2008, then re-sent a second and third time as necessary to non-respondents over the following 9 months. [23] With each wave of data collection, any missing baseline data were updated and any discrepancies between waves reconciled. Survey I, in Chinese (English if requested), included questions on activity level, developmental progress, as well as a food intake frequency questionnaire. Given that this was the first postal survey in this cohort, we used a limited number of food frequency questions, similar to ones used successfully in previous Hong Kong studies. [24], [25] After pilot testing, the food frequency questions included nine foods (dairy products, fruit, vegetables, soy products, fish, seafood, meat, egg and ice cream) and seven drinks (milk, tea, water, fruit juice, soy milk, soft drink and milkshake) which are commonly consumed in Hong Kong and vary among primary school students. [26].

**Figure 1:**
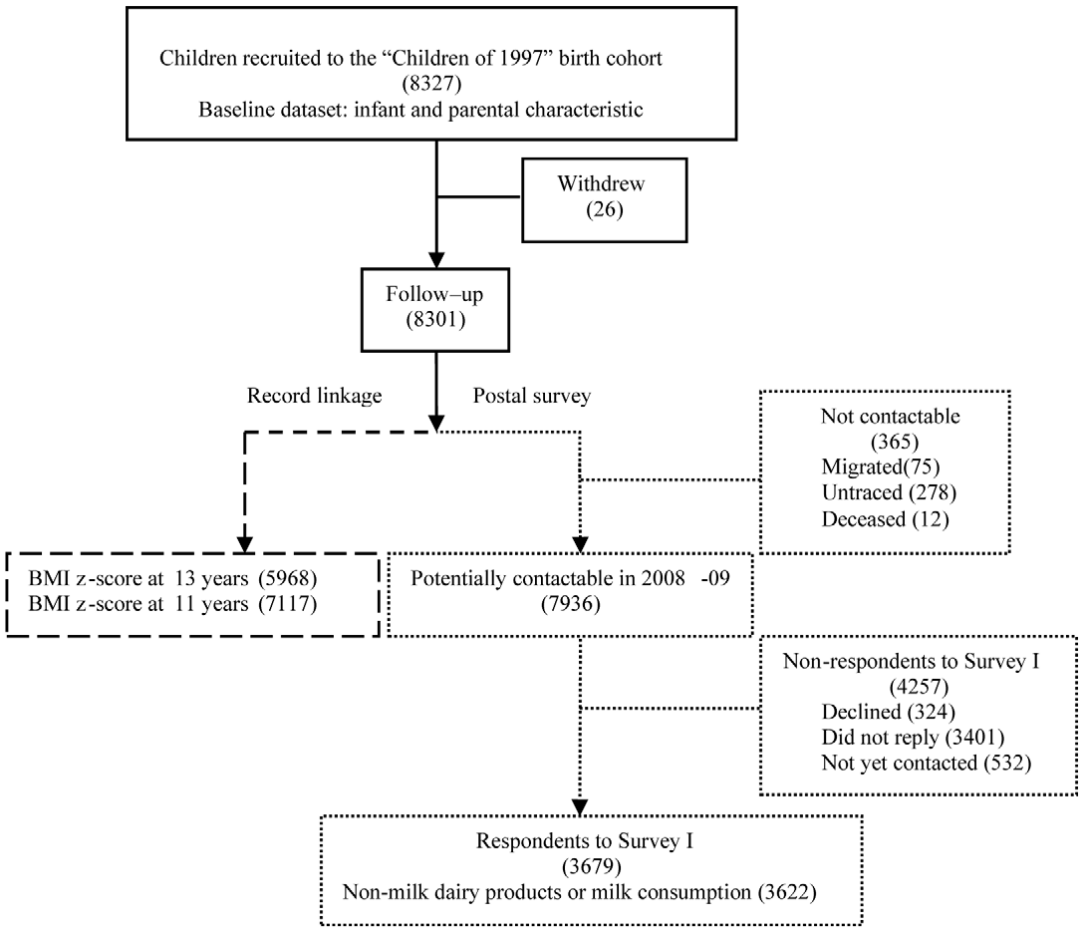
Hong Kong’s “Children of 1997” birth cohort recruitment, resurrection and follow-up (as at August 2011).

As shown in Figure 1, of the original 8,327 cohort members, as of 31^st^ August 2011, 26 had permanently withdrawn from the study. Of the remaining 8,301 children, 5,968 had height and weight measurement at about 13 years. 7,936 were potentially contactable in 2008–9 for Survey I, whilst 75 had migrated without trace, 278 were untraceable (probably migrated or dead) and 12 were known to be dead. Of these 7,936, 3,679 responded to Survey I, of whom 98% provide non-milk dairy product and milk consumption.

### Exposure

The primary exposures were frequency of non-milk dairy products (excluding milk, e.g. cheese/yogurt) consumption and the frequency of milk (e.g. cow’s milk/milk powder) consumption at about 11 years, categorized as “none during the past week” (by collapsing the two response categories of “never consumes”, “none in the last week”), “1–3 times during the past week”, “4–6 times during the past week”, and daily (incorporating “once a day”, “2–3 times a day” and “4 or more times a day”).

To validate the food frequency questions in Survey I, we used a pilot study for which 40 families took part. The examination included anthropometric measurements, blood pressure, bone mineral density, fat mass, muscle mass. Daily dairy product consumption was positively related to bone mineral density (whole body but without head, 0.08 g/cm^2^, 95% confidence interval 0.02 to 0.15) adjusted for sex, mother’s birthplace and highest parental education, suggesting at least some validity of the self-reported food frequency questions.

### Outcomes

The primary outcome was adiposity at about 13 years proxied by age and sex-specific BMI *z*-score relative to the 2007 World Health Organization (WHO) growth standard. [27] Not all height and weight measurements were exactly at age 13 years so we used the last measurement available after 12 years (mean age 12.9±0.4 years, range from 12.0 to 13.6, mean duration of follow-up 1.5±0.5 years). We interpolated the WHO growth standard on a daily scale using the “akima” package [28], [29] in R (R Development Core Team, Vienna, Austria version 2.11.1). [30] The secondary outcome was waist hip ratio (WHR) using self-reported waist and hip circumferences from Survey I.

### Missing Data

Among the 5,968 adolescents who had a BMI measurement at about 13 years of age, 51.5% had missing data for non-milk dairy products or milk consumption. Over 90% had infant characteristics and information on social-economic position (SEP) including birth weight, birth order, breastfeeding, second hand smoke exposure, maternal age at birth, mother’s birthplace (Mainland China or Hong Kong), highest parental education, occupation, household income and housing type (public housing or private flat). Multivariable logistic regression showed that sex, breastfeeding, second hand smoke exposure, maternal age and mother’s birthplace were related to missingness of our exposure. Given the missing data are not missing completely at random (MCAR), a complete case analysis might be biased. [31] We assumed the missing mechanism for our exposure is missing at random (MAR), rather than missing not at random (MNAR), because the non-response to Survey I was not likely to be directly associated with dairy product consumption, nor with unobserved variables, because we have detailed information about key attributes such as SEP for almost all cohort members. In this situation, multiple imputation (MI) reduces bias and improves efficiency (reduces standard errors). [31] In Survey I respondents, we found that sex, birth order, maternal age, mother’s birthplace, highest parental education, household income and housing type were associated with non-milk dairy products and milk consumption. We predicted missing values of exposures and confounders based on a flexible additive regression model with predictive mean matching [32] incorporating data on the primary outcome (BMI *z*-score at 13), [33] and all variables related to missingness or dairy product consumption. We imputed missing values 50 times using the “Hmisc” package in R. [34] We checked the distribution of the imputed data with the observed data and no obvious problem with the imputation process was found. We analyzed the 50 complete datasets separately and summarized the results into single estimated beta-coefficients with confidence intervals adjusted for missing data uncertainty. [35] For comparison we also carried out an available case analysis, i.e., pairwise deletion of observations with missing data. As a check for MI, we also repeated the analysis with combination of inverse probability weighting (IPW) and MI (IPW/MI). [36]

### Statistical Analysis

Multivariable linear regression models were used to examine the adjusted association of non-milk dairy products and milk consumption with BMI *z*-score and WHR. Confounders considered were sex, birth order, maternal age at birth, mother’s birthplace, highest parental education, household income, physical activity, vegetable, fruit and soft drink consumption. To illustrate the possible effect of socio-economic confounders, physical activity and food consumption, we present three models. Model 1 adjusted for sex, BMI z-score at 11 years (baseline BMI), birth order and maternal age. Model 2 additionally adjusted for mother’s birthplace, highest parental education, income and the interaction of mother’s birthplace and highest parental education, because we have previously shown that the association of parental education with BMI varies with mother’s birthplace. [18] Model 3 adjusted for all confounders mentioned. Further adjustment for other measures of socio-economic position (parental occupation), birth weight, breastfeeding, pubertal stage and other food consumption (fish, seafood, meat, soy milk, tea, water, etc.) did not change the estimates (data not shown). We also assessed whether the association varied with sex from the heterogeneity across strata and the significance of interaction terms. Although this is a large sample which we would expect to be robust to any deviations from the normality assumption in linear regression, we used residual plots and Q-Q plots to check the assumption and also repeated the analysis with a non-parametric method using re-sampling.

## Results

Of the 3,679 respondents who completed Survey I, 27.7% of the adolescent did not consume non-milk dairy products at all during the past week, 40.3% consumed 1–3 times during the past week, 11.7% consumed 4–6 times during the past week, while 20.3% consumed them daily. For milk consumption, 34.3% of the children did not consume at all during the past week, 34.4% consumed 1–3 times during the past week, 9.5% consumed 4–6 times during the past week and 21.8% consumed them daily. Of the 3,679 respondents, 3,084 had self-reported measurements on waist and hip circumferences. Table 1 shows that, in the available case analysis, those children with lower birth order, higher maternal age, mothers born in Hong Kong, more educated parents, or higher household income were more likely to consume non-milk dairy products or to drink milk. Physical activity, vegetable and fruit consumption were also positively associated with non-milk dairy products and milk consumption.

**Table 1:** Characteristics by non-milk dairy products and milk consumption for 3,679 adolescents from Hong Kong’s “Children of 1997” birth cohort (column %, available case analysis).

	Dairy products consumption during past week						Milk consumption during past week					
Characteristics	*n*	None	1–3 times	4–6 times	Daily	*P-value*	*n*	None	1–3 times	4–6 times	Daily	*P-value*
**BMI z-score at 11years**												
Mean (SD)		0.21(1.3)	0.24(1.3)	0.21(1.2)	0.28(1.2)	0.625		0.24(1.3)	0.22(1.3)	0.26(1.3)	0.26(1.3)	0.893
**Sex**												
Female	1,862	55.1	50.0	53.4	48.0	0.013	1,859	55.2	52.4	49.0	45.3	<0.001
Male	1,760	44.9	50.0	46.6	52.0		1,752	44.8	47.6	51.0	54.7	
**Birth order**												
1	1,694	46.4	46.3	47.9	54.7	0.008	1,648	46.7	45.0	53.1	52.9	0.001
2	1,464	42.7	43.9	40.5	36.6		1,461	41.7	43.9	38.3	39.7	
≥3	355	10.9	9.8	11.6	8.7		358	11.6	11.1	8.6	7.4	
**Maternal age at birth**												
≤24	328	11.4	9.2	9.6	6.6	0.007	328	10.6	9.2	9.2	7.8	0.196
25–29	1,103	33.3	32.2	29.0	28.4		1,094	31.5	32.3	32.6	28.2	
30–34	1,408	36.6	40.1	42.5	43.0		1,409	38.9	40.2	40.7	41.8	
≥35	680	18.7	18.5	18.9	22.0		678	18.9	18.3	17.5	22.2	
**Mother’s birthplace**												
Mainland China orelsewhere	1,323	44.7	35.4	29.9	32.1	<0.001	1,310	40.4	35.2	32.2	34.0	0.003
Hong Kong	2,285	55.3	64.6	70.1	67.9		2,288	59.6	64.8	67.8	66.0	
**Highest parental education**												
≤Grade 9	1,028	34.4	28.8	22.6	22.7	<0.001	1,028	33.5	28.5	24.2	22.3	<0.001
Grade 10–11	1,548	43.6	43.3	32.1	40.8		1,542	43.5	44.8	41.4	40.4	
≥Grade 12	1,046	22.0	27.9	35.3	36.5		1,041	24.0	26.7	34.4	37.3	
**Household income per head in quintiles (mean±SD)**												
1^st^ (HK$1751±413)	584	22.6	17.8	17.4	13.2	<0.001	582	21.0	18.4	15.0	14.9	<0.001
2^nd^ (HK$2856±325)	623	23.8	19.0	16.5	15.7		620	21.8	19.8	16.7	16.0	
3^rd^ (HK$4362±556)	636	18.1	22.2	16.8	19.2		630	20.3	20.4	20.3	17.5	
4^th^ (HK$6822±886)	672	19.3	21.0	20.7	23.2		668	20.3	20.8	21.3	21.8	
5^th^ (HK$14850±16050)	696	16.2	20.1	28.6	28.6		696	16.6	20.6	26.7	29.8	
**Physical activity**												
<1 hour a day	2,281	73.9	72.1	66.0	67.1	0.003	2,278	71.5	73.1	67.9	67.5	0.042
≥1 hour a day	941	26.1	27.9	34.0	32.9		939	28.5	26.9	32.1	32.5	
**Vegetable consumption during past week**												
None	69	3.0	1.9	1.2	0.9	<0.001	69	2.0	1.6	1.5	2.5	<0.001
1–3 times	329	9.7	10.9	9.9	4.4		324	10.3	10.0	7.3	6.1	
4–6 times	416	12.0	13.1	13.5	6.7		410	12.9	12.6	11.4	7.3	
Daily	2,792	75.3	74.1	75.4	87.9		2,790	74.8	75.8	79.8	84.1	
**Fruit consumption during past week**												
None	131	5.2	3.4	2.1	2.9	<0.001	132	4.5	2.5	3.5	4.3	<0.001
1–3 times	972	30.6	28.6	25.1	19.8		966	30.8	26.2	23.0	23.5	
4–6 times	667	17.9	20.7	24.6	11.3		656	17.3	21.8	23.6	11.7	
Daily	1,841	46.3	47.4	48.2	66.0		1,843	47.4	49.5	49.9	60.5	
**Soft drink consumption during past week**												
None	1,185	37.8	30.1	29.4	34.3	<0.001	1,190	36.1	29.7	30.5	35.2	<0.001
1–3 times	1,827	48.0	53.8	50.4	49.0		1,819	48.1	54.8	51.0	48.1	
4–6 times	338	7.5	10.3	11.9	8.7		339	8.5	10.4	13.2	7.7	
Daily	244	6.7	5.8	8.3	8.0		242	7.3	5.1	5.3	9.0	

Residual plots and Q-Q plots for linear regression models showed no violation of constant variance or normality assumptions. Re-sampling also produced very similar results (data not shown). Table 2 shows that, using imputed data for 5,968 adolescents with BMI *z*-score, neither non-milk dairy products nor milk consumption at 11 years was prospectively associated with BMI *z*-score at about 13 years, adjusted for sex, BMI z-score at 11 years, birth order and maternal age (Model 1), additionally adjusted for mother’s birthplace, highest parental education, household income and the interaction of mother’s birthplace and highest parental education (Model 2), or further adjusted for physical activity, vegetable, fruit and soft drink consumption (Model 3). There was no evidence that any associations varied with sex (all *p*-values>0.3).The available case analysis produced similar results and is shown in Table S1. IPW/MI also produced very similar results (data not shown).

**Table 2:** Mean difference in BMI *z*-score at about 13 years of age by non-milk dairy products and milk consumption in 5,968 adolescents from Hong Kong’s “Children of 1997” birth cohort (multiple imputation).

Consumptionduring past week		BMI *z*-score											
		Model 1^a^				Model 2^b^				Model 3^c^			
	*n*	β^d^	95% CI			β^d^	95% CI			β^d^	95% CI		
Dairy products													
None	1,633	Reference				Reference				Reference			
1–3 times	2,494	0.01	−0.04	to	0.06	0.01	−0.04	to	0.06	0.01	−0.04	to	0.06
4–6 times	717	0.02	−0.05	to	0.10	0.02	−0.05	to	0.10	0.02	−0.05	to	0.10
Daily	1,124	−0.0002	−0.07	to	0.07	−0.0004	−0.07	to	0.07	0.001	−0.07	to	0.07
*P-value for trend*		0.908				0.908				0.886			
Milk													
None	2,067	Reference				Reference				Reference			
1–3 times	2,110	0.001	−0.05	to	0.05	0.002	−0.05	to	0.05	0.003	−0.04	to	0.05
4–6 times	555	−0.01	−0.08	to	0.06	−0.01	−0.08	to	0.06	−0.01	−0.08	to	0.06
Daily	1,236	−0.01	−0.07	to	0.05	−0.01	−0.07	to	0.05	−0.01	−0.07	to	0.05
*P-value for trend*		0.597				0.667				0.655			

a Model 1 adjusted for sex, BMI z-score at 11 years, birth order and maternal age.

b Model 2 additionally adjusted for mother’s birthplace, highest parental education, interaction of mother’s birthplace and education.

c Model 3 additionally adjusted for physical activity, vegetable, fruit and soft drink consumption.

d Mean difference in BMI *z*-score: at age of 13.0 years, 1 unit of change in BMI *z*-score is approximated to 2.9 kg/m^2.^

CI = confidence interval.

In 3,084 children who reported both waist and hip circumferences, neither non-milk dairy products nor milk consumption at about 11 years was associated with WHR cross-sectionally in any model (Table 3).

**Table 3:** Mean difference in waist hip ratio at about 11 years of age by non-milk dairy products and milk consumption in 3,084 adolescents from Hong Kong’s “Children of 1997” birth cohort (available case analysis).

Consumptionduring past week		Waist hip ratio											
		Model 1^a^				Model 2^b^				Model 3^c^			
	*n*	β^d^	95% CI			β^d^	95% CI			β^d^	95% CI		
Dairy products													
None	846	Reference				Reference				Reference			
1–3 times	1,253	−0.01	−0.02	to	0.003	−0.01	−0.02	to	0.01	−0.003	−0.01	to	0.01
4–6 times	360	0.0001	−0.01	to	0.01	0.003	−0.01	to	0.02	0.01	−0.01	to	0.02
Daily	625	−0.03	−0.02	to	0.01	0.001	−0.01	to	0.01	−0.001	−0.02	to	0.01
*P-value for trend*		0.868				0.731				0.887			
Milk													
None	1,065	Reference				Reference				Reference			
1–3 times	1,068	−0.01	−0.02	to	0.002	−0.01	−0.02	to	0.003	−0.01	−0.02	to	0.004
4–6 times	295	−0.01	−0.02	to	0.01	−0.004	−0.02	to	0.01	−0.004	−0.02	to	0.01
Daily	656	−0.01	−0.02	to	0.01	−0.005	−0.02	to	0.01	−0.005	−0.02	to	0.01
*P-value for trend*		0.421				0.535				0.586			

a Model 1 adjusted for sex, birth order and maternal age.

b Model 2 additionally adjusted for mother’s birthplace, highest parental education, household income, interaction of mother’s birthplace and education.

c Model 3 additionally adjusted for physical activity, vegetable, fruit and soft drink consumption.

d Mean difference in waist hip ratio.

CI = confidence interval.

## Discussion

In this large, population-representative birth cohort of Chinese adolescents, neither non-milk dairy products nor milk consumption (at about 11 years) was related to BMI *z*-score prospectively (at about 13 years) or waist hip ratio cross-sectionally (at about 11 years). There was no evidence of different associations by sex. These findings are inconsistent with findings in some Western observational studies, [4], [5], [6], [7], [13] but more consistent with most RCTs evaluating the effect of either dairy product or milk supplementation. [14], [15], [16].

Our analysis has some limitations. First, in common with most other studies, dietary consumption was obtained by self report which may be subject to recall error. Misclassification of dairy product consumption is likely to bias any estimates towards the null. However, reported dairy product intake was validated against bone mineral density in a subgroup of children. Second, we do not have contemporaneous dietary information at other ages. There may be different effects of dairy product consumption during infancy or early childhood. However, that does not negate the value of understanding the role of dairy product consumption in early adolescence when lifelong dietary habits may be formed. Third, we proxied adolescent obesity by BMI, which cannot differentiate between lean mass and body fat. However, we found a similar pattern for WHR in a subset. Fourth, we did not have information on total energy intake, nor did we differentiate types of milk (skimmed, low fat or full cream milk). However, the estimates were unchanged by additional adjustment for other foods (model 3 compared with model 2) suggesting that residual confounding by other dietary factors is unlikely. Fifth, lactase persistence is uncommon in southern Chinese, so some adolescents drinking milk may have had difficulty digesting it. However, this would, if anything, have biased the findings towards an association of milk consumption with lower BMI, which we did not see. Conversely, milk consumption may largely correspond to lactase persistence. However, there is no evidence that this directly affects BMI, although it may lead to higher calorie intake and higher BMI.

Our findings are consistent with 3 RCTs in adolescents evaluating the effect of either dairy product or milk supplementation, [14], [15], [16] which found no effect on body fat, although these trials mainly concerned girls. Our observations concerned both sexes and found no difference by sex.

Our study is inconsistent with findings in some observational studies [1], [2], [4], [5], [6], [7], [13] but more consistent with some others [8], [9], [10], [11], [12] from Western populations. Cross-sectional studies are subject to reverse causality. [1], [2] People who are overweight or obese may consume less dairy product or milk to avoid dairy fat. Moreover, observational studies are open to residual confounding. In long-term developed countries low socio-economic position (SEP) is associated with both childhood adiposity, [37], [38] and low dairy product consumption. [19], [39] However, not all of these studies adjusted for SEP, which may explain the mixed findings in Western populations. In our population, SEP is not clearly associated with child adiposity. [18] Our estimates were unchanged by additionally adjusted for SEP (model 2 compared with model1), suggesting confounding by SEP is unlikely. This suggests that the inverse relation of dairy product or milk consumption with adiposity found in Western countries may be due to residual confounding by SEP.

Alternatively, it has been suggested dairy products may reduce body fat via the effects of calcium. [40] It is possible that sources of dietary calcium vary between our population and Western settings, such that dairy products are a more important source of and marker of calcium intake in Western than in Asian populations. Currently the evidence from trials of the role of calcium in body fat is equivocal. [41], [42] Nevertheless, we cannot rule out the possibility that dairy products may be protective against overweight and obesity because they are an important dietary source of calcium. However, if that is the case, public health policy would best focus on low calorie sources of calcium rather than dairy products.

### Conclusions

In this population based Chinese birth cohort, neither non-milk dairy products nor milk consumption was associated with lower adolescent BMI *z*-score prospectively or with WHR cross-sectionally, with or without adjustment for SEP, physical activity and other food consumption. The negative association observed in Western settings may be due to socially patterned confounding by SEP. Our study demonstrates the role of evidence from different social contexts in confirming or refuting empirically driven hypotheses from long-term economically developed populations.

## Supporting Information

Table S1 **Mean difference in BMI** ***z*** **-score at about 13 years of age by non-milk dairy** products and milk consumption in 3,622 adolescents from Hong Kong’s “Children of 1997” birth cohort (available case analysis, i.e. without multiple imputation).
(DOCX)
