# Supplementary material for: The Role of Dairy Products and Milk in Adolescent Obesity: Evidence from Hong Kong’s “Children of 1997” Birth Cohort
Source: PLoS One. 2012 Dec 20;7(12):e52575. doi: 10.1371/journal.pone.0052575 (PMC3527590; doi:10.1371/journal.pone.0052575)
Supplement: Table S1 — Mean difference in BMI z -score at about 13 years of age by non-milk dairy products and milk consumption in 3,622 adolescents from Hong Kong’s “Children of 1997” birth cohort (available case analysis, i.e. without multiple imputation). (DOCX) [file pone.0052575.s001.docx]

Table S1 Mean difference in BMI *z*-score at about 13 years of age by non-milk dairy products and milk consumption in 3,622 adolescents from Hong Kong’s “Children of 1997” birth cohort (available case analysis, i.e. without multiple imputation)

| Consumption  during past week |  | **BMI *z*-score** | | | | | | | | | | | |
| --- | --- | --- | --- | --- | --- | --- | --- | --- | --- | --- | --- | --- | --- |
|  |  | **Model 1^a^** | | | | **Model 2^b^** | | | | **Model 3^c^** | | | |
|  | *n* | β^d^ | 95% CI | | | β^d^ | 95% CI | | | β^d^ | 95% CI | | |
| Dairy products |  |  |  |  |  |  |  |  |  |  |  |  |  |
| None | 803 | Reference | |  |  | Reference | |  |  | Reference | |  |  |
| 1-3 times | 1,183 | 0.02 | -0.03 | to | 0.07 | 0.04 | -0.01 | to | 0.10 | 0.04 | -0.02 | to | 0.10 |
| 4-6 times | 339 | 0.04 | -0.04 | to | 0.11 | 0.05 | -0.02 | to | 0.13 | 0.06 | -0.02 | to | 0.14 |
| Daily | 572 | 0.001 | -0.07 | to | 0.07 | 0.03 | -0.05 | to | 0.10 | 0.04 | -0.04 | to | 0.12 |
| *P-value for trend* |  | 0.818 |  |  |  | 0.420 |  |  |  | 0.249 |  |  |  |
|  |  |  |  |  |  |  |  |  |  |  |  |  |  |
| Milk |  |  |  |  |  |  |  |  |  |  |  |  |  |
| None | 1,003 | Reference | |  |  | Reference | |  |  | Reference | |  |  |
| 1-3 times | 997 | 0.01 | -0.04 | to | 0.06 | -0.01 | -0.06 | to | 0.05 | -0.02 | -0.07 | to | 0.04 |
| 4-6 times | 282 | -0.004 | -0.08 | to | 0.07 | -0.05 | -0.13 | to | 0.04 | -0.05 | -0.14 | to | 0.03 |
| Daily | 615 | -0.01 | -0.07 | to | 0.06 | -0.02 | -0.09 | to | 0.05 | -0.05 | -0.12 | to | 0.03 |
| *P-value for trend* |  | 0.629 |  |  |  | 0.348 |  |  |  | 0.128 |  |  |  |

a. Model 1 adjusted for sex, BMI z-score at 11 years, birth order and maternal age

b. Model 2 additionally adjusted for mother’s birthplace, highest parental education household income, interaction of mother’s birthplace and education

c. Model 3 additionally adjusted for physical activity, vegetable, fruit and soft drink consumption

d. Mean difference in BMI *z*-score: at age of 13.0 years, 1 unit of change in BMI *z*-score is approximated to 2.9 kg/m^2^

CI=confidence interval
